# Survey of the Effect of Streptokinase on Ventricular Repolarization by Examining the QT Dispersion in Patients With Acute Myocardial Infraction in Seyed-Al-Shohada Hospital, Urmia

**DOI:** 10.5539/gjhs.v6n7p74

**Published:** 2014-09-18

**Authors:** Rahimi Darabad B., Vatandust J., Pourmousavi Khoshknab M. M., Seyed Mohammad Zad M. H.

**Affiliations:** 1 Reproductive Health Research Center, Urmia University of Medical Science, Urmia, Iran; 2 Urmia University of Medical Sciences - Seyedoshohada Heart Hospital, Urmia, Iran

**Keywords:** acute myocardial, patients, Iran

## Abstract

Cardiovascular events are the most common cause of morbidity and mortality throughout the world and myocardial infarction is the most common cause of these accidents. Myocardial infarction impairs the mechanical and electrical activity of the heart that these disorders predispose the patient to cardiac arrhythmias including ventricular tachycardia. QT dispersion is an important parameter to evaluate the heterogeneity of ventricular repolarization that minimal and the maximum interval is QTc in 12-lead EKG. In this study, 200 patients with the diagnosis of acute myocardial infraction with ST-segment elevation were hospitalized and treated with streptokinase. Patient records were extracted from the medical records department. EKG was studied before receiving streptokinase, an hour after receiving streptokinase and 4 days later for calculating and comparing QTd. It was concluded that QTd mean in EKG one hour after receiving streptokinase is decreased compared to pre-operation but this decline is not statistically significant. QTd mean in EKG day 4 after MI is slightly increased compared to the baseline, which is not statistically significant.

## 1. Introduction

QT dispersion is the minimal and the maximum interval of QTc in 12-lead EKG and indicates the heterogeneity of ventricular repolarization. Impaired electrical activity after heart failure cause heterogeneity of ventricular repolarization and predispose the patient to cardiac arrhythmias. This heterogeneity of ventricular repolarization and impaired electrical activity is due to ischemia or myocardial infarction that is created by the blockage of the arteries that supply the myocardium or coronary artery. Treatment with streptokinase that is a fibrinolysis drug increases the likelihood of opening a blocked coronary artery and re-established the blood flow. With reperfusion of ischemic areas, heart’s electrical and mechanical activity is improved and the possibility of heterogeneity of ventricular repolarization and arrhythmias is decreases. Cardiovascular events are the most common cause of morbidity and mortality throughout the world. Myocardial infarction that is caused by complete or incomplete obstruction of feeder vessels (coronary) is the most common cause of these accidents. In the U.S nearly a million patients infected acute myocardial infarction, and more than one million patients with possible acute myocardial infarction are admitted to the coronary care unit (CCU) (Zipes, Braunwald, & Libby, 2001). Acute coronary syndrome include: 1-Acute myocardial infarction associated with elevated segment ST (STEMI) segment 2-Acute myocardial infarction without elevation of ST (NSTEMI) segment 3-Unstable angina (UA). Patients with acute myocardial infarction with ST-segment elevation undergoing reperfusion of blood flow in the arteries feeding the heart, this re-perfusion occurs either by fibrinolytic agents such as streptokinase or by using PCI (percutaneous coronary intervention or percutaneous coronary intervention) (Szymański, Rezler, & Budaj, 1999). Ischemic myocardium alters the electrical and mechanical activity of the myocardium. Disturbance in the electrical activity of the heart results in heterogeneity of ventricular repolarization activity that is induced arrhythmias (Day, McComb, & Campbell, 1990). Re-perfusion after ischemia may profoundly alter the function of the heart. Mediations of this problem are oxygen radicals and neutrophils. An important parameter to evaluate the heterogeneity of ventricular repolarization is electrocardiographic QT dispersion (Dr. Sargiz Youssefian, 2008). QT Dispersion (QTd) means the difference between the maximum and minimum QT interval obtained from 12-lead ECG (Giedrimiene, 2003). The longer the QTd is the greater heterogeneity of the ventricular repolarization and predisposed arrhythmias. The first technique to evaluate heterogeneous repolarization is the potential monophasic action. Using this invasive technique, local heterogeneity in potential action in different parts of the cardiac action was shown and heterogeneous repolarization for ventricular arrhythmias is affirmed. Thus, it was found that an increase in the QT interval by different reasons and increase in dispersion is associated with potential monophasic action. Body Surface Mapping was second technique that would examine the quality of ventricular repolarization, however, non-invasive, was time-consuming and tedious. It should be noted that in a healthy person, the longest QT interval is on the left and behind the chest and shortest is in the lower right side of the chest can be drawn by the electrodes. Investigate QTd after acute myocardial infarction indicates heterogeneity in repolarization of hurt parts with healthy parts of the heart put the patient at the risk of poor prognosis and odds of ventricular tachycardia (Enar et al., 2001; Bonnar et al., 1999). Studies show that taking drugs such as beta blockers and sotalol or use of PCI is reduced QTd (Segerson et al., 2008). In the acute phase of MI in patients with anterior MI show QT Interval longer than inferior MI suggesting a change in the extent of myocardial repolarization. Increased QT mechanism is changes in autonomic and electrical conductivity by irritation or ischemia. Patients with long QT syndrome and Torsade de point are also show the lack of coordination in their ventricular repolarization. The relationship between QTd and extent of myocardial ischemia has been reported. Roukema et al. also demonstrated a direct correlation between the prolongation of the QT interval and myocardial ischemia (Jensen et al., 2005). Repolarization become longer in myocardial infarction and thus QT in ECG is shown longer (Bonow et al., 2011). Previous studies have demonstrated that QTd was significantly, immediately after exercise testing in patients with coronary stenosis, (greater than 50%) was higher than in patients without stenosis.

One of the most important treatments in acute myocardial infarction associated with ST segment elevation is fibrinolytic agents. Mechanism of fibrinolytic drugs containing streptokinase, urokinase, tenecteplase and anistreplase is through changing plasminogen into fibrinolytic enzyme activator, plasmin. Plasmin streptokinase complex is not inhibit by A2-antiplasmin and therefore can effectively reduce fibrin clot. Streptokinase and urokinase through activating fibrinolytic system that decreases anti-plasmin a2 and can cause systemic effects (Vaarala, 1998). According to the bacterial source of streptokinase, it has antigenic properties and most people have antibodies that are related to their previous streptococcal infection. Prescribing streptokinase stimulates formation of neutralizing anti-streptokinase and antibodies and by neutralizing prescribed streptokinase dose impeded the fibrinolytic effect of streptokinase (Bili et al., 2000). Streptokinase causes transient blood pressure loss in many patients and in some of them would also create allergic reactions including rash, fever, and bronchospasm (Enar, 2001). Another rare complication is cerebral hemorrhage. Given that ventricular repolarization activity after acute myocardial infarction plays an important role in dangerous arrhythmias, the factors that are involved in changing ventricular (QTd), including reperfusion are important. Therefore, in this study the effect of streptokinase that is the major fibrinolytic used in Iran on ventricular repolarization activity (QT) is studied to evaluate the role of ventricular repolarization activity and formation of arrhythmias after acute myocardial infarction. In previous foreign studies of streptokinase effect and PCI on QT dispersion is investigated. In Iran PCI effect on QT dispersion has been investigated but not studied streptokinase and other fibrinolytic effects and this study is the first study to investigate the streptokinase effect on QT dispersion in Iran.

## 2. Methods

This is a retrospective study in 2013 in Seyed-al-shohada Subspecialty Heart Hospital, Urmia. A total of 200 patients (based on previous studies) admitted to Seyed-al-shohada Hospital, Urmia where they confirmed the diagnosis of acute myocardial infarction and treated with streptokinase were enrolled. In this study, all patients admitted to hospital with acute myocardial infarction treated with streptokinase were enrolled. Records of these patients were excluded from the medical records department and studied. Data include demographic characteristics (age, gender, etc.) have been extracted and photographs were taken from ECG before and one hour after receiving streptokinase and 4 days after acute myocardial infarction. Inclusion criteria: Patients with acute myocardial infarction associated with ST-segment elevation by clinical symptoms, laboratory findings (elevated cardiac troponin I) and ECG changes were confirmed and treated with streptokinase.

Exclusion criteria:

· 1 - Electrolyte disorders.
· 2 - Absence of sinus rhythm.
· 3 - Cardiac conduction block.
· 4 - Inability to study ECG at least at the 8-lead.
· 5 - Drugs influence on QTd such as digitalis, antidepressants, antipsychotics.

Images taken from the ECG of patients after magnification have been reviewed and QT was calculated manually. Methods to calculate QT: the beginning of the Q wave and the onset of QRS complex to the end of T wave was calculated as the QT interval. In cases where the U wave was present in the lowest point of the curve between the T and U wave was considered as the QT interval. QTc was calculated by Bazzet formula. The longest and the shortest QTc interval were calculated in ECGT12 Lydia and the difference was considered as QTd. Data analysis was performed using statistical software SPSS20 (Kaveh & Dalfard, 2014).

## 3. Findings

Of 200 patients in the study after inclusion and exclusion criteria, 40 patients were excluded:

2 electrolyte imbalance (hyponatremia).

5 cases the absence of sinus rhythm in the first EKG.

10 cases block types (4 cases LBBB, 5 cases RBBB and one case CHB).

5 cases taking digitalis.

7 cases not being able to check in at least 8-lead.

9 cases the lack of EKG before and after streptokinase. One case died.

One case although had ST-T changes and were received streptokinase but had normal cardiac enzymes (UA)

Of the 160 patients enrolled, 122 patients were male and 38 were female. 105 patients aged greater than or equal to 65 years and 55 patients were older than 65 years. 53 patients had hypertension, 107 patients were free of hypertension. 24 patients were diabetic and 136 were non-diabetic patients. 72 patients were smokers and 88 were non-smoking patients. Site of myocardial infarction in 84 patients was anterior (ant), 54 patients were with acute inferior myocardial infarction (inf), 7 patients with lateral acute stroke (lat) and 15 patients had mixed stroke.

QTd1 minimum is zero and maximum value is 204.96 ms with a mean of 76.25 and standard deviation of 35.502.

QTd2 minimum is zero and maximum value is 194.03 ms with a mean of 70.93 and standard deviation of 27.98 ms.

QTd3 minimum is zero and maximum value is 244.95 ms with a mean of 76.69 and standard deviation of 47.81 ms.

QTd mean changes over time in the form of total QTd mean in EKG an hour after receiving streptokinase (QTd2) has decreased, but this reduction was not statistically significant (p= 0.831).

QTd3 mean compared to QTd2 has increased but compared to QTd1 had almost no change, and this change was not also statistically significant (p = 0.831).

**Figure 1:**
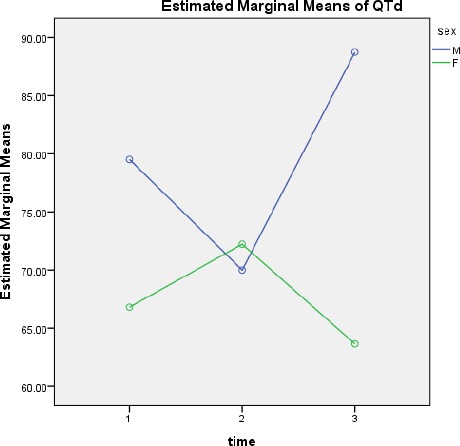
QTd mean changes over time according to gender

Compared QTd changes based on sex.

In male mean and standard deviation of QTd was QTd1 = 79.94 ± 37.68, QTd2 = 68.49 ± 39, QTd3 = 87.56 ± 50.06, respectively.

In female QTd1 = 67.83 ± 27.05, QTd2 = 73.74 ± 33.82, QTd3 = 62.05 ± 36.04. QTd changes over time between men and women is statistically significant (p = 0.048).

**Figure 2:**
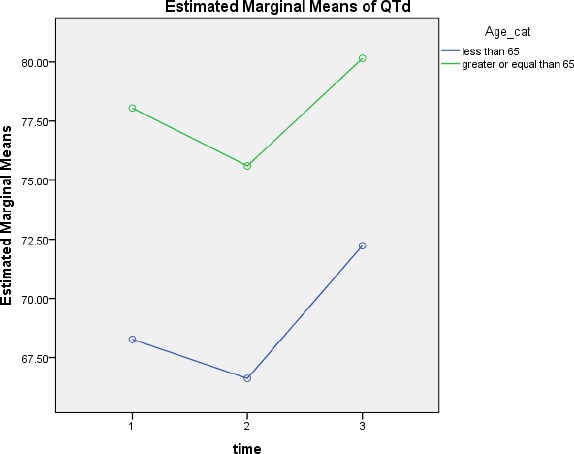
QTd changes over time according to age group

Age ≥ 65 years, the mean QTd1, QTd2 and QTd3 was, 25.18 ± 78.24, 45.60 ± 76.18, 50.54 ± 79.71, respectively and

Age less than 65 years the mean QTd1, QTd2 and QTd3 was, 35.48 ± 68.32, 32.62 ± 66.36, 46.57 ± 72.23, respectively. Difference between the two groups is statistically significant (p=0.015). However, QTd changes over time separately, there is no significant difference between the groups (p = 0.979).

**Figure 3:**
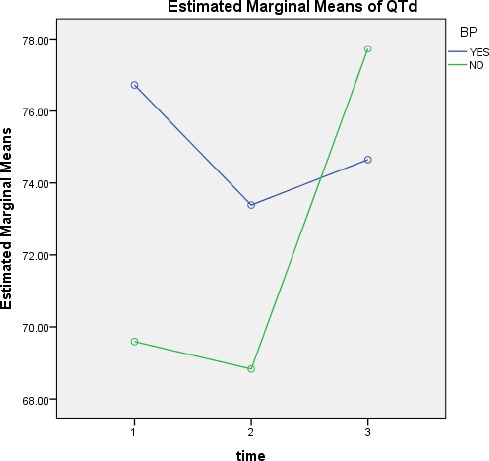
QTd mean changes over time according to normal or high blood pressure

In patients with hypertension QTd1, QTd2 and QTd3 was, 35.14 ± 76.86, 48.16 ± 73.71, 36.15 ± 74.18, respectively and in patients without hypertension QTd1, QTd2 and QTd3 was, 32.16 ± 69.73, 45.72 ± 68.93, 37.46 ± 77.89, respectively. QTd changes in patients with hypertension over time were not significant (p=0.56). However, in patients without hypertension, there was a significant difference between QTd2 and QTd3 (p=0.028). Between patients with and without hypertension, there was no significant difference (p=0.068).

**Figure 4:**
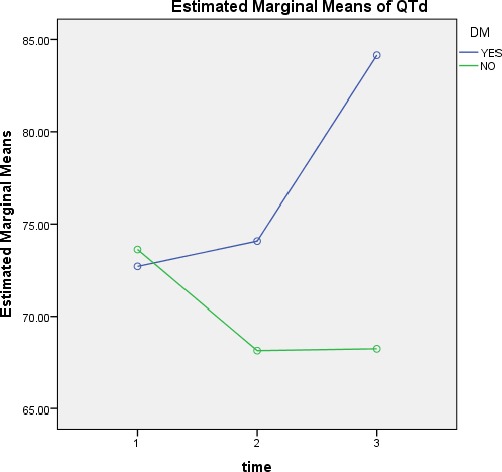
QTd mean changes based on time in diabetic and non-diabetic patients

QTd1, QTd2 and QTd3 in diabetics patients was, 39.17 ± 72.37, 42.15 ± 74.18, 32.11 ± 84.86, respectively and in patients without diabetes was, 33.65 ± 73.92, 25.29 ± 68.81, 39.41 ± 68.14, respectively.

QTd changes in diabetic and non-diabetic patients over time were not significant (p=0.078 and p=0.451).

QTd changes between diabetics and non-diabetics are also not significant (p=0.378).

**Figure 5:**
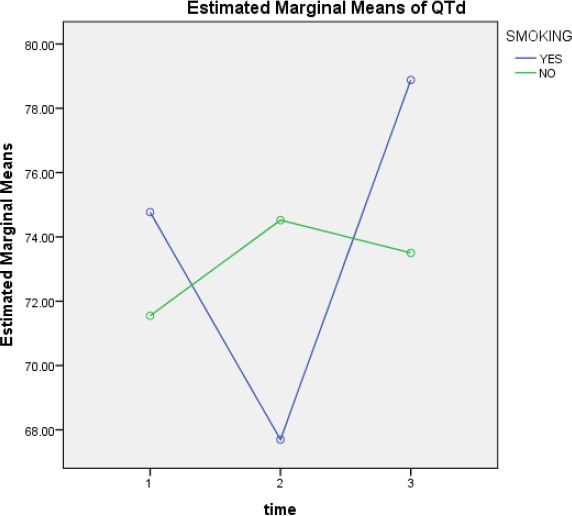
QTd mean changes over according to being a smoker or non-smoker

Smoking rates of QTd1, QTd2 and QTd3 were, 32.17 ± 74.78, 40.14 ± 67.90, 28.25 ± 79.26, respectively and in non-smoking patients QTd1, QTd2 and QTd3 was, 37.14 ± 71.86, 39.36 ± 74.66, 29.15 ± 73.80, respectively.

QTd changes over time in smokers, although the rate of QTd2 has declined compared to QTd1 and QTd3 compared to QTD2 and QTd1 has increased, but these changes were not significant (p=0.348).

QTd changes in the non-smokers was also not significant (p=0.741). Between smokers and non-smokers there was a significant difference (p=0.016).

**Figure 6:**
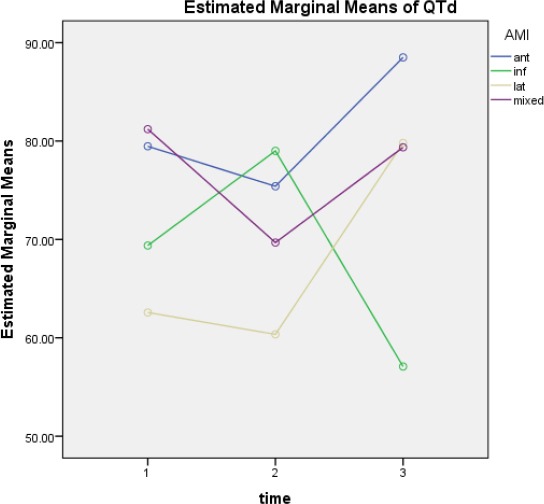
QTd mean changes over time based on the location of the heart stroke

QTd1, QTd2 and QTd3 in acute anterior myocardial infarction were 25.37 ± 79.91, 32 ± 75.14, 47 ± 88.11 ms, respectively.

QTd1, QTd2 and QTd3 in acute inferior infarction were 30 ± 69.27, 41 ± 78.22, 34 ± 56.86 ms, respectively.

QTd1, QTd2 and QTd3 in lateral acute stroke were 28 ± 62.75, 41 ± 60.11, 37 ± 78.86 ms, respectively.

QTd1, QTd2 and QTd3 in complex acute stroke were 25 ± 81.14, 40 ± 69.26, 32 ± 78.45 ms, respectively.

Between the anterior and inferior infarction, in the anterior QTd2 rate has reduced compared to QTd1 and QTd3 compared to QTd2 has increased, but in the inferior QTd2 rate has increased compared to QTd1 and QTd3 compared to QTd2 has been significantly reduced that as a result of the difference between anterior and inferior infarction was significant (p=0.0248).

QTd changes over time based on the location of acute stroke, the only significant difference is the inferior type (p=0.0311).

## 4. Discussion and Conclusion

In Nikoforos, Cavusoglu and Chanders study, the effect of PCI and fibrinolytic on QTd were assessed and compared. Outcome was such that both treatments were successful in reperfusion reduces QTd but the effect of PCI compared with fibrinolytic in reducing QTd is higher. In our study only examined the effect of streptokinase that QTd rate in EKG at one hour after treatment with streptokinase was decreased, but in EKG the fourth day after MI had a slight increase (Nikiforos, 2003, Cavusoglu et al., 2001, Chander et al., 2004). In a study by Neusa et al. on the effect of reperfusion in the blocked artery on QTd, the result was such that in the group with reperfusion was shown a significant decrease in blood flow p<0.001, but in the group without reperfusion QTd rate was increased tremendously p<0.001. In our study, reperfusion after treatment with streptokinase was not examined (Neusa et al., 2003). In Moreno et al. (Moreno, 1994) study on the effect of therapy on QTd was found that the successful fibrinolytic treatment (TIMI≥2) reduces QTd and JTd but has no effect on the QRS, but in our study the QTd rate an hour after receiving streptokinase was reduced but QTd rate 4 days after MI was increased. Reasons that can cause an increase or lack of decrease in QTd in our study may be stated as follows:

1)-Lack of reperfusion rate survey after treatment with streptokinase. If the reperfusion rate is specified and QTd rates of patients are compared based on reperfusion perhaps there may be a significant difference between the group with reperfusion and without reperfusion. Maybe the number of patients with reperfusion is lower than the patients without reperfusion that is increasing instead of decreasing QTd.

2)-The use of streptokinase as fibrinolytic therapy. In previous studies primarily alteplase is used as fibrinolytic, which its effect on opening blocked arteries is probably more than streptokinase.

3)-Errors in measurements and calculations. QT and RR intervals are done manually on the EKG. To reduce measurement error, EKG was examined after magnification and all measurements were performed by one person.

4)-Lack of timely use of streptokinase. In most of the previous studies the patients were included into the study when the interval between onset of symptoms and fibrinolytic prescription is less than 12 hours; however, in our study due to the lack of specification between onset of symptoms and hospital admission in the records, this criterion was not examined.

Nikoforos et al. study in 2003 compared the effects of fibrinolytic therapy and PCI in reducing QTd was concluded that PCI is more effective in reducing QTd p<0.001. Also, in this study changes in QTd in patients with and without hypertension were compared that there was no significant difference between them p=0.19. In our study, the amount of QTd in patients with hypertension compared with patients without hypertension was higher but the difference was not significant p=0.068 (Nikiforos et al., 2003). In Cavusoglu et al. study on patients with anterior stroke compared to inferior stroke QTd rate was higher. p=0.026 In the current study, in the anterior stroke QTd has decreased one hour after treatment and at the fourth day has increased. In the inferior stroke, QTd rate one hour after receiving streptokinase has increased and at the fourth day has reduced that the difference between these two groups is statistically significant, p = 0.024 Cavusoglu, 2001). In the Chanders et al. study between male and female sex in terms of reduced QTd after reperfusion, there was no significant difference. p=0.32 However, in our study in male patients QTd1 rate was higher than female patients and has reduced one hour after treatment with streptokinase and 4 days after MI has increased significantly. But in female patients QTd rate has increased one hour after treatment and has decreased on the fourth day. Difference between males and females was significant, p = 0.048 (Chander et al., 2004).

Mehta et al. had conducted a study (2003) to compare QTd rate and fibrinolytic effect in elderly patients and younger patients. QTd was measured manually at the time of admission, after fibrinolytic therapy and the second day after myocardial infarction in 36 patients with more or equal to 65 years and 36 patients aged less than 65 years. Conclusion was that: before treatment QTd rate in older people was more than young people (7.3 ± 76.6 ms vs. 7.5 ± 69.6 with the p-value <. 0001). After fibrinolytic therapy, there were also differences between the two groups (7.2 ± 69.6 ms vs. 8.4 ± 69.1 ms with the p-value <.001). On the second day after treatment QTd in the older group was8.2 ± 74.1ms and the young group was 9.1 ± 69 ms with the p-value = .001 (Mehta et al., 1994). In our study also QTd1, QTd2 and QTd3 rates in patients aged more or equal to 65 years was higher than in patients aged less than 65 years that this difference was statistically significant. In our study, QTd changes based on smoking and non-smoking of patients were also compared. In smokers QTd before treatment was higher than non-smokers. One hour after receiving streptokinase QTd has dropped and on the fourth day has increased, but in non-smokers QTd rate has increased one hour after treatment and on the fourth day had decreased but compared to primary QTd has increased but the difference is not statistically significant between the groups. p=0.348 in the previous studies QTd changes were not studied based on smoking and non-smoking patients. In the present study we compared QTd changes based on diabetic and non-diabetic patients that were not conducted in the previous studies. In non-diabetic patients QTd rate before treatment was slightly higher than diabetic patients, but there was not statistically significant difference. QTd rate in diabetic patients one hour after streptokinase therapy and on the fourth day has increased. QTd rate in non-diabetic patients one hour after receiving streptokinase and on the fourth day has decreased, but the difference is not statistically significant (p = 0.378).
